# Direct measurement of X-ray-induced heating of microcrystals

**DOI:** 10.1107/S1600577519003849

**Published:** 2019-05-14

**Authors:** Anna J. Warren, Danny Axford, Robin L. Owen

**Affiliations:** a Diamond Light Source, Harwell Science and Innovation Campus, Didcot OX11 0DE, UK

**Keywords:** X-ray-induced beam heating, fluorescence, cryocooling

## Abstract

Temperature control is a key aspect of macromolecular crystallography, with the technique of cryocooling routinely being used to mitigate X-ray-induced damage. Beam-induced heating could cause the temperature of crystals to rise above the glass transition temperature, greatly increasing the rate of damage. X-ray-induced heating of ruby crystals of 20–40 µm in size has been quantified non-invasively by monitoring the emission wavelengths of X-ray-induced fluorescence during exposure to the X-ray beam. For the beam sizes and dose rates typically used in macromolecular crystallography, the temperature rises are of the order of 20 K. The temperature changes observed are compared with models in the literature and can be used as a validation tool for future models.

## Abstract 2

X-ray-induced heating of microcrystals is quantified through the temperature-dependent shift in X-ray-induced fluorescence from ruby crystals.

## 1. Introduction   

X-ray-induced damage is an inevitable aspect of macromolecular crystallography at synchrotron sources (Garman, 2010 ▸). By far the most successful and widely implemented means of mitigating damage is to hold samples at ∼100 K using an open-flow nitrogen cryostat. A key tenet of this approach is the assumption that the crystal is held below the glass transition temperature of ∼130–140 K (Johari *et al.*, 1987 ▸; McMillan & Los, 1965 ▸; Sartor *et al.*, 1994 ▸) during data collection (Weik *et al.*, 2000 ▸). Above this temperature radical species are mobile within the crystal and, as a result, the rate at which radiation damage occurs greatly increases. During a diffraction experiment, radical species are generated within the crystal through the X-ray-induced radiolysis of water. This results in the formation of solvated electrons and hydroxyl radicals (Klassen, 1987 ▸). While electrons and holes are mobile down to 77 K (Jones *et al.*, 1987 ▸; Symons, 1995 ▸), the mobility of hydroxyl radicals increases significantly between 115 K (Zakurdaeva *et al.*, 2005 ▸) and 130 K (Symons, 1999 ▸). The energy deposited by the X-ray beam during data collection causes the temperature of the sample to rise above the nominal holding temperature of 100 K. As storage rings and beamlines evolve, the increasing fluxes and flux densities available mean that this beam-induced heating could cause the temperature of crystals to increase above the glass transition point. This would result in a greatly accelerated rate of damage and reduced crystal lifetimes. Beam-induced heating is also a concern in room-temperature macromolecular crystallography (MX) as reaction rates increase as temperature increases; a rule of thumb from the Arrhenius equation is that reaction rates double for every 10 K increase. Unless beam heating can be reduced, or outrun, any gains made by collecting data more rapidly using more intense beams may be limited owing to increased reaction and diffusion rates.

Despite the potentially deleterious implications of beam-induced heating in synchrotron-based MX, a relatively limited amount of research has been carried out in this area. Helliwell (1984 ▸) described a simple adiabatic model of beam-induced heating. Later models take into account both conduction of heat within the sample and heat transfer away from the sample by the surrounding gas stream (Nicholson *et al.*, 2001 ▸; Kriminski *et al.*, 2003 ▸; Kuzay *et al.*, 2001 ▸; Mhaisekar *et al.*, 2005 ▸). In all cases it was shown that, as conductive heat transfer is more efficient than convection, temperature variations within the sample are expected to be small in comparison to the temperature difference between the sample and the surrounding gas stream.

In order to directly measure X-ray beam-induced heating, an experimental approach was taken in which a thermal imaging camera was used to observe the heat rise within 1 and 2 mm diameter glass beads and validate theoretical predictions (Snell *et al.*, 2007 ▸). In this study, it was shown at a third-generation synchrotron source that the temperature rise within a flow of nitrogen gas was of the order of 10–15 K, *i.e.* not enough to exceed the glass-transition temperature. However, the continuous development of beamlines and synchrotron sources means that beam sizes are continually becoming smaller, with a concomitant increase in flux density. Furthermore, a 1 mm glass bead has a volume some four or five orders of magnitude greater than a typical protein crystal, which may easily be less than 50 µm in its longest dimension.

More recently, Mykhaylyk *et al.* (2017 ▸) reported noncontact luminescence lifetime cryothermometry for the *in situ* measurement of different protein-sample mounts within the vacuum vessel of beamline I23 at Diamond Light Source. A small piece of scintillating material (∼100–200 µm) was used, where the luminescent decay time could be measured, and the temperature of the crystal could be derived using calibration curves. They were then able to test how effectively differing loop types kept the crystal cool at the sample position, observing a temperature range of 60–110 K with the gonio­meter temperature held at 40 K. This method of temperature measurement proved interesting as the scintillating material has a volume more similar to that of a protein crystal and, like the infra-red experiments described above, the measurements were taken without the need for any physical connections to the crystal.

Here, we report an investigation into the effect of heating on micrometre-sized crystals on the microfocus beamline I24 at Diamond Light Source. The (laser and X-ray induced) fluorescence of ruby crystals provides a convenient means of quantifying the X-ray-induced temperature rise in small, protein crystal-sized, samples. As in the experiments by Snell and co-workers and Mykhaylyk and co-workers referenced above, this approach has the advantage that no cables or other physical connections between the sample and probe that might transfer heat to or from the sample are required. Ruby has the additional advantage that it is a well characterized system and temperature-dependent changes in fluorescence have been the subject of many systematic studies. The most common means of inducing ruby fluorescence is with a green laser (Syassen, 2008 ▸), but X-ray-induced fluorescence can also be exploited. In both cases, as the temperature of ruby increases the wavelength of the fluorescence peaks also increases (Syassen, 2008 ▸; Ragan *et al.*, 1992 ▸; McCumber & Sturge, 1963 ▸). For the experiments reported here this temperature-dependent line shift provides a convenient micrometre-sized temperature probe.

Experimental measurement of beam-induced heating in ruby crystals allows the validation of beam-induced heating models and hence the prediction of whether new sources and beamlines will cause the temperature of protein crystals to increase beyond tolerable limits.

## 2. Materials and methods   

### 2.1. Beamline and spectrometer parameters   

Data were collected on beamline I24 at Diamond Light Source using a beam size of 20 µm × 20 µm (dimensions are full width at half-maximum; Gaussian profile, uncollimated beam) and X-rays of energy 12.8 keV with an incident flux of 1.19 × 10^12^ photons s^−1^ and 9.2 keV with an incident flux of 3.18 × 10^12^ photons s^−1^. Photon fluxes were measured using a silicon PIN diode (Owen *et al.*, 2009 ▸). These flux densities allowed dose rates of up to 1.5 MGy s^−1^ (12.8 keV) and 7.4 MGy s^−1^ (9.2 keV) to be realized in ruby samples. Absorbed doses were calculated using *RADDOSE*-3*D *(Zeldin *et al.*, 2013 ▸). The dose values quoted and used below are the average diffraction-weighted dose reported by this program. In *RADDOSE*-3*D* the small-molecule option was utilized, allowing zero solvent content to be specified.

**Figure 1:**
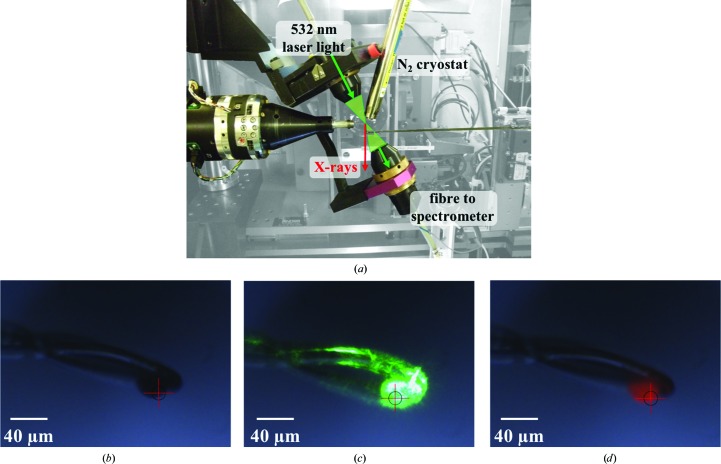
(*a*) Experimental setup on beamline I24, showing the sample position with the spectrometer installed and the cryostream in place to control the temperature of the experiments. (*b*, *c*, *d*) Views of a 40 µm ruby sphere (*b*) mounted within a nylon loop at the sample position, (*c*) illuminated with the 532 nm laser and (*d*) when exposed to the X-ray beam, demonstrating the fluorescence of the ruby.

Emission spectra were collected using mirrored lenses (Bruker) mounted in an off-axis geometry [Fig. 1 ▸(*a*)]. The lenses were positioned so the focus of each coincided with the intersection of the goniometer rotation axis and X-ray beam [Fig. 1 ▸(*b*)]. Absorption was monitored over the 560–830 nm wavelength range using a Shamrock 303 imaging spectrograph (Andor). The spectrograph was calibrated to better than 0.01 nm using a mercury argon lamp (Ocean Optics) and the calibration was confirmed by cross-comparison of spectra collected using a second spectrograph with a fixed grating.

**Figure 2:**
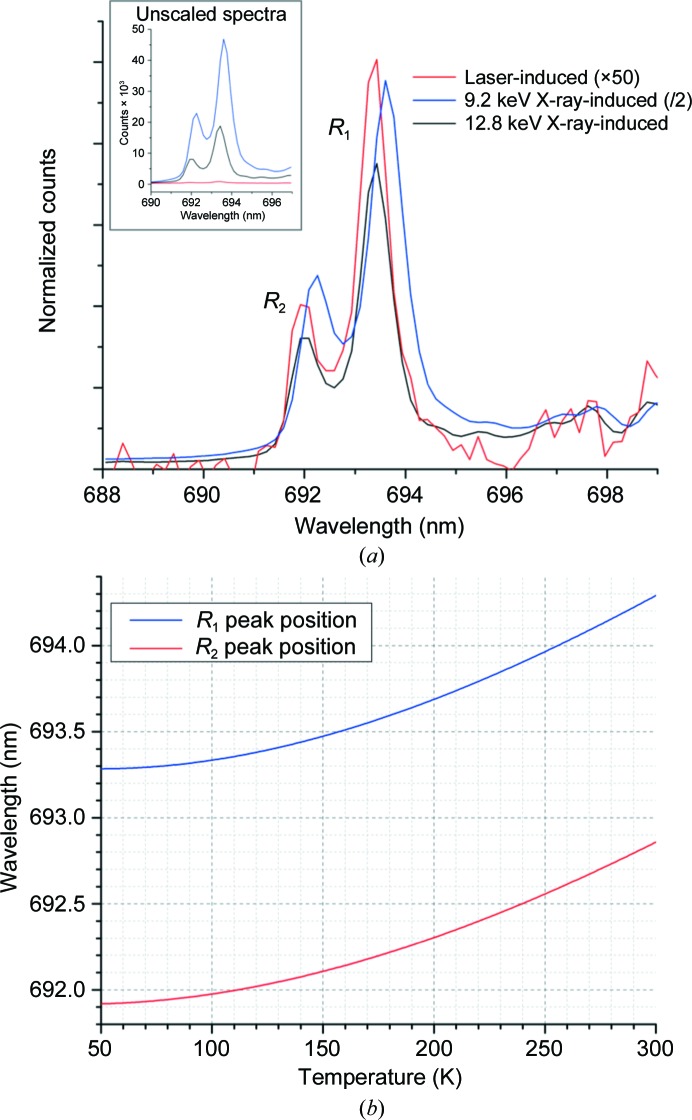
(*a*) Laser- and X-ray-induced fluorescence of ruby, showing peak shifts and the relative intensity of laser- and X-ray-induced spectra. In addition to being red-shifted, the *R*_1_ and *R*_2_ peaks become broader at higher temperatures. The inset shows the unscaled spectra. (*b*) Calibration curve showing the position in wavelength of the ruby *R*_1_ and *R*_2_ fluorescence peaks obtained from (2) and (3). Note that the cubic function has a stationary point at 50 K (*R*_1_) and 40 K (*R*_2_), so temperatures below this would result in a peak with the same wavelength at a higher temperature. Temperatures below 80 K were not investigated as part of this study.

For laser-induced fluorescence, a 532 nm laser was used for excitation [shown in Fig. 1 ▸(*c*)]. The laser power at the sample position was 13 µW (measured using a Thorlabs power meter). Each measurement was taken for 0.2 s with an accumulation of five images, except where time-evolution studies were carried out, in which 5, 10 and 20 ms images (one accumulation) were used. In the setup described, the X-ray-induced fluorescence [shown in Fig. 1 ▸(*d*)] was an order of magnitude more intense than that induced by the laser [Fig. 2 ▸(*a*)].

### 2.2. Sample preparation   

Ruby spheres were purchased from DiamondAnvils (https://www.diamondanvils.com) with diameters varying between 10 and 50 µm. Spheres were mounted on nylon loops using as little Fomblin Y oil (Sigma–Aldrich) as possible and were placed directly on the beamline and cooled in an open-flow nitrogen cryostat held at 100 K. Ruby is aluminium oxide doped with a small amount of chromium: the asymmetric unit of ruby is (Al_0.33_Cr_0.00333_)O_0.5_ in space group 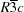 with unit-cell parameters *a* = 4.75, *c* = 12.99 Å. The aluminium and chromium share a third occupancy on a special position, and the oxygen has half occupancy, also on a special position.

For all data collected the nitrogen cryostat was set to the default flow rate of 10 l min^−1^. For experiments using a helium cryostat it was not possible to set the helium flow rate to a specific value. The flow was set so it was approximately the same as that of nitrogen based on the helium consumption of the cryostat given by the manufacturer (Cryo Industries of America) and the gas-expansion coefficient of helium, and visually keeping the flow rate constant over the duration of the experiment.

### 2.3. Data analysis   

Laser-induced ruby *R*_1_ and *R*_2_ fluorescence peaks are shown in Fig. 2 ▸(*a*) and are best described by a Lorentzian profile (Ragan *et al.*, 1992 ▸). *OriginPro* was used to fit the experimental data with a double Lorentzian of the form
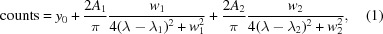
where *y*_0_ is an offset and *A_i_* is the area, *w_i_* is the width and λ_*i*_ is the centre of each peak. The wavelength dependence of the *R*_1_ and *R*_2_ fluorescence peaks as a function of temperature, *T*, was determined from the functions given by Ragan *et al.* (1992 ▸). The peak positions in cm^−1^ are given by
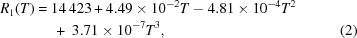


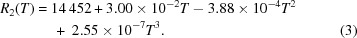
Calibration curves relating wavelength (1/wavenumber) to temperature derived from (2) and (3) are shown in Fig. 2 ▸(*b*). As *R*_1_ is the larger peak, the position of this peak was used to calculate the temperature of the crystal. The difference in temperature calculated using the position of *R*_1_ versus that calculated from the position of *R*_2_ was typically less than 3 K. For brevity, we refer to the temperature measured and calculated in this way as the ‘fluorescence temperature’ below, and all ruby crystal temperatures refer to the observed fluorescence temperature.

## 3. Results   

**Figure 3:**
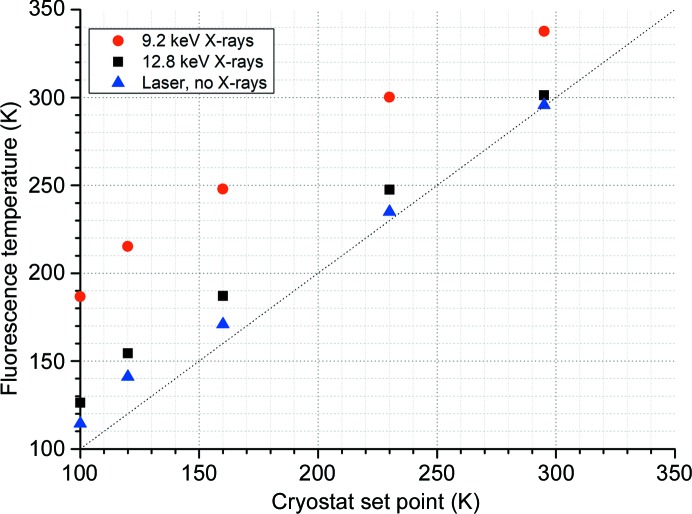
Optical laser (no beam) and X-ray fluorescence temperature of a 40 µm ruby crystal as a function of the cryostat set point. The X-ray beam size was uncollimated 20 µm × 20 µm, Gaussian with incident flux 3.18 × 10^12^ and 1.19 × 10^12^ photons s^−1^ at 9.2 and 12.8 keV, respectively (as detailed in Section 2.1).

Emission spectra were collected from two ruby crystals, the first with a diameter of 20 µm (*i.e.* matching the beam size) and the second with a diameter of 40 µm. Laser-induced emission spectra were relatively weak [Fig. 2 ▸(*a*)], and data collection at different laser powers resulted in no discernible peak shift. The crystal temperatures recorded using the laser alone were therefore taken to be representative of the temperature of ruby crystals held in the nitrogen flow in the absence of external heating. In the absence of X-rays, the difference between the fluorescence temperature and the nominal set point of the nitrogen cryostat was ∼15 K with the cryostat set to 100 K. Note that this refers to the fluorescence temperature determined from optical laser-induced fluorescence, when probe-induced heating of the sample is expected to be almost zero. The difference between the fluorescence temperature and the cryostat setpoint was observed to decrease to zero as the cryostat setpoint temperature increased from 100 K to room temperature (Fig. 3 ▸). This difference may originate from an error in the cryostat calibration or imperfect positioning of the sample in the gas stream. All subsequent data refer to beam-induced temperature changes and thus are unaffected by this offset.

**Figure 4:**
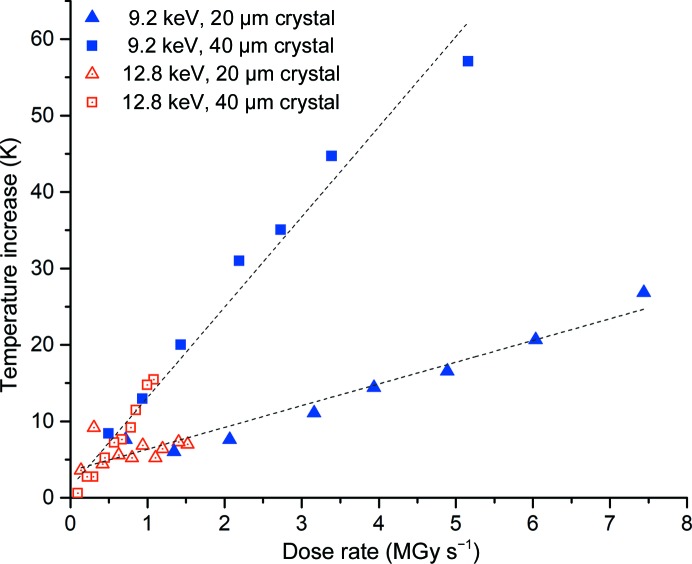
The effect of dose rate on the temperature increase of ruby crystals. Blue and red points indicate data collected at 9.2 and 12.8 keV, respectively. Data collected with the crystal size matched to the beam are shown as triangles; squares indicate that the crystal was larger than the beam.

**Figure 5:**
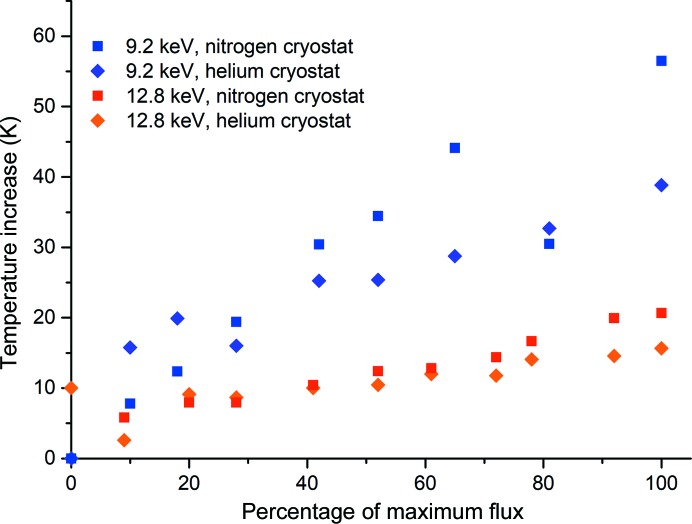
Effect of the cryostat gas on the change in temperature. To allow direct comparison of data collected at 9.2 and 12.8 keV, temperature changes are plotted against the percentage of the maximum flux rather than the flux density or dose. The flux densities and the resulting absorbed doses are the same as those shown in Fig. 4 ▸. Data were collected from a 40 µm diameter ruby sphere.

The observed change in temperature as a function of dose rate is shown in Fig. 4 ▸. It can be seen that the rate of change is independent of energy for a given crystal size and is a function of absorbed dose. The large X-ray cross-section of ruby means that extremely high dose rates are reached at both X-ray energies. The dose rates at 12.8 keV are more representative of those realized in protein crystallography (<5 MGy s^−1^). At this energy, for the 40 µm ruby crystal the maximum increase in temperature is 15 K, while for the 20 µm ruby crystal the maximum temperature rise is 5 K. Linear fits to the data are overlaid, and the intercepts (*i.e.* the temperature rise induced at a dose rate of 0 Gy s^−1^) are 1.3 ± 0.8 K for the 20 µm crystal and 3.5 ± 0.6 K for the 40 µm crystal. The deviation of these (and the intercepts in Fig. 5 ▸) from 0 K reflects the uncertainty in the measurements and could arise from experimental error in several parameters such as X-ray flux and beam size, crystal size and fluorescence wavelength.

The effect of the gas used to cool the sample is shown in Fig. 5 ▸. To allow direct comparison of data collected at 9.2 and 12.8 keV, data are plotted as a function of the maximum flux shown in Fig. 4 ▸. The use of helium reduces the X-ray-induced temperature rise. In the case of 9.2 keV X-rays the temperature increase at maximum flux is reduced from 58 to 39 K, while the change is reduced from 21 to 15 K for 12.8 keV X-rays: a reduction of ∼30% in each case. The use of helium as a cryogen clearly significantly reduces the X-ray-induced temperature increases and this could be particularly useful when extremely brilliant sources are used and samples are subjected to extremely high dose rates. However, any gains from keeping crystals below the glass transition temperature have to be tensioned against the ease of use and ubiquity of nitrogen cryostats, the finite availability and cost of helium, and the extremely short lifetime of protein crystals in such intense X-ray beams.

**Figure 6:**
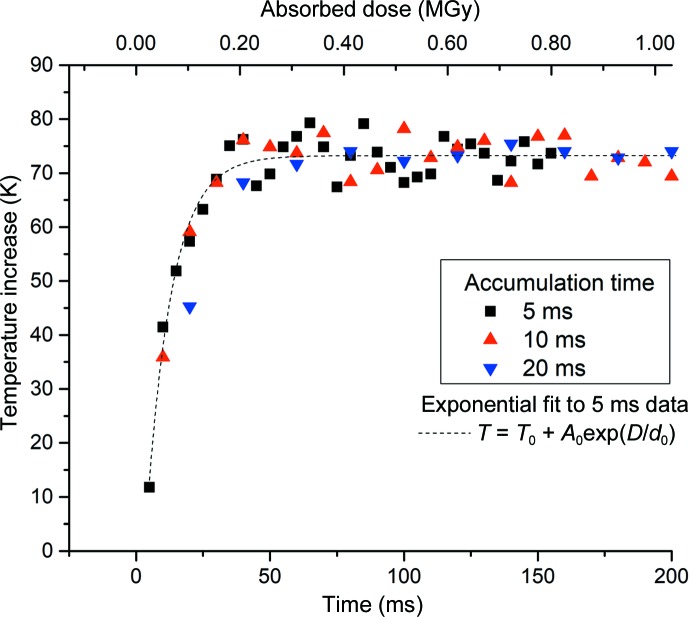
Rate of change of fluorescence temperature induced by 9.2 keV X-rays in a 40 µm ruby crystal. Data were collected with a 5, 10 and 20 ms accumulation time: the 5 ms data points exhibit more noise but greater temporal resolution. The initial rate of change of temperature (over the first 10 ms) is >6000 K s^−1^, with a steady-state temperature being reached after ∼40 ms. Data were collected at an incident dose rate of 5.1 MGy s^−1^.

In order to quantify the time required for samples to reach a steady-state temperature in the X-ray beam, the wavelength shift of ruby fluorescence was recorded as a function of time (Fig. 6 ▸). It can be seen that the initial rate of change of temperature is extremely large (>6000 K s^−1^), but a steady-state fluorescence temperature is reached relatively quickly after only ∼30 ms (∼150 kGy). An exponential fit of the form temperature rise = *T*_0_ + *A*_0_ exp(−dose/*d*_0_), where *T*_0_, *A*_0_ and *d*_0_ are constants, was fitted to the data with the shortest integration time (5 ms). In this case, the quantity *d*_0_ was 49 ± 5 kGy, meaning that the dose required for half of the final temperature rise to be reached was just 34 kGy (∼6 ms).

## 4. Discussion and conclusions   

Several models for X-ray-induced heating of samples have been proposed; here, we briefly summarize them and compare the calculated temperature rises of ruby with those observed experimentally. The most basic model of beam-induced temperature changes is an adiabatic model, *i.e.* ignoring any heat exchange. In this case, the temperature rise can be calculated by dividing the energy absorbed by the mass and the specific heat capacity of the sample (the symbols used in the following equations are defined in Table 1 ▸). As the dose is the energy absorbed per unit mass, *Q*/*m* can be replaced by the absorbed dose *D*,
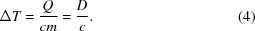
We refer to this as the ‘basic model’. Temperature rises for the samples and beam parameters used in this work have been calculated and are shown in Table 2 ▸. It can be seen from the table that the basic model poorly describes the heating of crystals by X-rays, with temperature rises of hundreds of Kelvins predicted in tens of milliseconds, reflecting the limitations of such a simple model.

Kuzay *et al.* (2001 ▸) demonstrated that for all but the briefest time periods it is necessary to consider the convection of heat away from the sample when determining the temperature change induced by X-rays: crystals are heated internally by X-rays and externally cooled at the surface by the gas stream. This was also taken into account by Nicholson *et al.* (2001 ▸), although the finite element analysis model described was not available to us and so is not compared with the data collected here. The Kuzay model of X-ray-induced temperature change within a sample was refined by Kriminski *et al.* (2003 ▸), who showed that the temperature change at the surface of a sample relative to that of the bulk gas stream surrounding it is given by
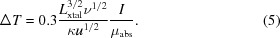
The assumption made that the absorbed power *P*_abs_ ≃ *IV*/μ_abs_, where *V* is the volume of the sample, can be used to reformulate this in terms of the more familiar absorbed energy per unit mass or dose, *D*, rather than the X-ray energy flux per square metre:
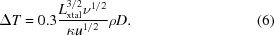
Again, the symbols used are defined in Table 1 ▸. We refer to (6) as the KKT model below. It should be noted that in this equation the absorbed dose *D* is the absorbed dose per second, or dose rate, rather than the total dose absorbed over the duration of the experiment. It can be seen from Table 2 ▸ that the temperature rises predicted by the KKT model agree well with the fluorescence temperature of the ruby crystals. Both the predicted and observed temperature rises follow the same trends, with larger crystals showing larger temperature rises and a helium cryostat providing more efficient cooling. The temperature increases are overestimated by a factor of ∼2 in the case of ruby crystals in a nitrogen stream irradiated at 9.2 keV, but are somewhat closer (within ∼6 K) in all other cases. The reason for this discrepancy is not clear, but it may result from underestimation of the nitrogen flow or overestimation of the crystal size in these experiments. As an example, if the size of the 40 µm crystal was overestimated by 5 µm then the predicted steady-state temperature increase would reduce from 121 to 101 K.

Heat transfer within a crystal is more efficient than heat transfer through the surrounding gas stream. It might therefore be expected that increasing the size of the crystal beyond that of the X-ray beam would result in a ‘fin effect’ where unirradiated regions of the crystal act as a heat sink. In this case the temperature rise for cube-shaped crystals would be reduced by a factor of (*L*_beam_/*L*_crystal_)^1/2^ (Kriminski *et al.*, 2003 ▸). The absence of this effect can be accounted for by the Gaussian profile of the beam: there is significant X-ray intensity in the tails of the beam, beyond the full width at half-maximum quoted beam size, resulting in the observed and predicted larger temperature rise in 40 µm ruby crystals. The beam intensity also varies along the beam path through the crystal. The X-ray attenuation lengths in ruby at 9.2 and 12.8 keV are ∼110 and 290 µm, respectively (calculated using *RADDOSE-3D*). Thus, in the case of a 40 µm ruby crystal the beam intensity decreases by 30% (9.2 keV X-rays) or 13% (12.8 keV X-rays), which may result in the overestimation of modelled temperature rises. The study by Snell *et al.* (2007 ▸) was unique in that it was able to resolve temperature changes both temporally and spatially. In cases such as this, when the beam intensity varies significantly across the sample and when the sample size is also significantly larger than the X-ray beam (as was the case for the 1 and 2 mm glass beads used by Snell and co-workers), significant steady-state temperature gradients may result within the sample and ‘single-temperature methods’ such as X-ray-induced fluorescence can offer only a partial view of beam-induced heating.

The agreement between the predicted and observed temperature changes in ruby gives confidence in the predicted temperature increases in protein crystals. The temperature increases predicted (Table 2 ▸) at 12.8 keV are relatively small (<3 K). It should be noted, however, that the beam sizes and fluxes used in this study are (now) relatively modest: a threefold increase in flux has recently been realized at I24, while the beam can also be more tightly focused. If a protein crystal matched to a top-hat beam of 5 µm × 5 µm is exposed to 3 × 10^12^ photons s^−1^ 12.8 keV X-rays the predicted temperature rise increases threefold to 9 K (dose rate of 28 MGy s^−1^). While this should still result in a protein crystal remaining below the glass transition temperature, if heavy atoms such as selenium are added to the crystal composition then the absorbed dose increases significantly, as does the predicted temperature rise (to 26 K). Decreasing the energy of the incident X-rays means higher dose rates are more easily realized, with a concomitant increase in beam-induced heating. As the X-ray-induced temperature change in crystals is proportional to the absorbed dose (the KKT model described above), this may provide motivation for data collection at higher energies when extremely intense X-ray beams are used. For small samples this gain may be further increased by the reduction in absorbed dose resulting from photoelectron escape (Cowan & Nave, 2008 ▸). In Fig. 3 ▸ an offset of ∼15 K between the fluorescence temperature and the nominal set point of the nitrogen cryostat was observed. If a temperature offset owing to inefficient cryocooling or alignment of a cryostat is combined with beam-induced heating then it is clear that increased rates of damage may result and the crystal lifetimes in conventional nitrogen-cooled cryocrystallography may be significantly less than expected.

In conclusion, we have shown that ruby crystals and their fluorescence can be used as a convenient method for measuring X-ray-induced temperature changes. These results correlate well with the KKT model. With the beam parameters used here this model predicts X-ray-induced temperature rises of <3 K in protein crystals, with the result that they should remain well below the glass transition temperature. At current and near-future synchrotron beamlines significantly higher flux densities can be achieved, however, and the heating of samples to temperatures in excess of this remains a concern and challenge for microfocus MX.

## Figures and Tables

**Table 1:** Physical parameters relevant to sample X-ray beam-induced heating and their abbreviations and values as used in this work Kinematic viscosities and thermal conductivities were taken from Kriminski *et al.* (2003 ▸).

Abbreviation	Quantity	Units and typical value where applicable
*c*	Specific heat capacity	5 × 10^2^ J K^−1^ kg^−1^ (protein)
		7.5 × 10^2^ J K^−1^ kg^−1^ (ruby)
ρ	Density of sample	1.2 × 10^3^ kg m^−3^ (protein)
		3.98 × 10^3^ kg m^−3^ (ruby)
μ_abs_	X-ray absorption coefficient	8.93 mm^−1^ (ruby at 9.2 keV)
		3.27 mm^−1^ (ruby at 12.8 keV)
*L*_xtal_	Size of crystal	m
*ν*	Kinematic viscosity of gas stream	1.9 × 10^−6^ J K^−1^ kg^−1^ m^2^ s^−1^ (nitrogen, 100 K)
		20 × 10^−6^ J K^−1^ kg^−1^ m^2^ s^−1^ (helium, 100 K)
*κ*	Thermal conductivity of cryogen	9.8 × 10^−3^ W m^−1^ K^−1^ (nitrogen, 100 K)
		7.6 × 10^−3^ W m^−1^ K^−1^ (helium, 100 K)
*u*	Velocity of cryogen	∼3.3 m s^−1^ (estimated from flow rate; Section 2.2)

**Table 2:** Comparison of predicted and observed X-ray beam-induced heating The models used are described in the main text. The temperature change calculated using the basic model assumes 30 ms of exposure to X-rays (the approximate time taken to reach a steady-state temperature; Fig. 6 ▸). The KKT model and observed temperature rises are steady-state temperatures. The dose rate is calculated from the average diffraction-weighted dose reported by *RADDOSE*-3*D*.

					Δ*T* (K)		
Sample	Energy (keV)	Sample size (µm)	Cryogen	Dose rate (MGy s^−1^)	Basic model	KKT model	Observed
Ruby	9.2	20	Nitrogen	7.44	296	61.6	27
		40	Nitrogen	5.16	205	120.6	58
			Helium	5.16	205	50.5	39
	12.8	20	Nitrogen	1.52	60	12.5	7
		40	Nitrogen	1.08	43	25.2	21
			Helium	1.08	43	10.5	15
Protein	9.2	20	Nitrogen	2.30	138	5.7	—
		40	Nitrogen	1.68	101	11.8	—
			Helium	1.68	101	5.0	—
	12.8	20	Nitrogen	0.458	27	1.4	—
		40	Nitrogen	0.336	138	2.4	—
			Helium	0.336	138	1.0	—
